# Poverty, Food Insufficiency and HIV Infection and Sexual Behaviour among Young Rural Zimbabwean Women

**DOI:** 10.1371/journal.pone.0115290

**Published:** 2015-01-27

**Authors:** Sophie J. S. Pascoe, Lisa F. Langhaug, Webster Mavhu, James Hargreaves, Shabbar Jaffar, Richard Hayes, Frances M. Cowan

**Affiliations:** 1 Faculty of Epidemiology & Population Health, London School of Hygiene & Tropical Medicine, London, United Kingdom; 2 Centre for Sexual Health & HIV Research, Royal Free & University College Medical School, London, United Kingdom; 3 Centre for Sexual Health and HIV AIDS Research (CeSHHAR) Zimbabwe, Harare, Zimbabwe; Institute of Infectious Diseases and Molecular Medicine, SOUTH AFRICA

## Abstract

**Background:**

Despite a recent decline, Zimbabwe still has the fifth highest adult HIV prevalence in the world at 14.7%; 56% of the population are currently living in extreme poverty.

**Design:**

Cross-sectional population-based survey of 18–22 year olds, conducted in 30 communities in south-eastern Zimbabwe in 2007.

**Objective:**

To examine whether the risk of HIV infection among young rural Zimbabwean women is associated with socio-economic position and whether different socio-economic domains, including food sufficiency, might be associated with HIV risk in different ways.

**Methods:**

Eligible participants completed a structured questionnaire and provided a finger-prick blood sample tested for antibodies to HIV and HSV-2. The relationship between poverty and HIV was explored for three socio-economic domains: ability to afford essential items; asset wealth; food sufficiency. Analyses were performed to examine whether these domains were associated with HIV infection or risk factors for infection among young women, and to explore which factors might mediate the relationship between poverty and HIV.

**Results:**

2593 eligible females participated in the survey and were included in the analyses. Overall HIV prevalence among these young females was 7.7% (95% CI: 6.7–8.7); HSV-2 prevalence was 11.2% (95% CI: 9.9–12.4). Lower socio-economic position was associated with lower educational attainment, earlier marriage, increased risk of depression and anxiety disorders and increased reporting of higher risk sexual behaviours such as earlier sexual debut, more and older sexual partners and transactional sex. Young women reporting insufficient food were at increased risk of HIV infection and HSV-2.

**Conclusions:**

This study provides evidence from Zimbabwe that among young poor women, economic need and food insufficiency are associated with the adoption of unsafe behaviours. Targeted structural interventions that aim to tackle social and economic constraints including insufficient food should be developed and evaluated alongside behaviour and biomedical interventions, as a component of HIV prevention programming and policy.

## Introduction

Zimbabwe has seen a significant decline in HIV prevalence, driven in part by behaviour change [1–5]. Yet it still has one of the largest and most sustained HIV epidemics globally. In 2011 HIV prevalence among youth aged 15–24 years was 7.7% among females and 2.9% among males. [6].

The relationship between poverty and HIV infection in sub-Saharan Africa is complex and has been the subject of much debate over recent years [7–18]. A disproportionate number of those affected live in the poorer areas of the world, although HIV is not always concentrated among the poorest populations in these areas. [15, 19–24] The relationship between socio-economic position and HIV may also change over time with higher HIV prevalence shifting from the urban wealthy into impoverished and more rural populations. [10, 14, 25–28]

Socio-economic factors may act as a distal determinant of infection. Poor women are often economically dependent on men [11, 14, 29]. The need for economic support may partly drive earlier marriage and may make it difficult for young women to insist on safer sexual practices. Food insufficiency can also drive the adoption of high-risk behaviours.[18, 30, 31] It can also effect nutritional pathways which can affect both vertical and horizontal transmission of HIV.[18] The poorest women may have little choice but to adopt behaviours that put them at risk of infection, including transactional and intergenerational sex, earlier marriage, and relationships that expose them to violence and abuse. [7, 9, 11–14, 30–35] Poorer and less-educated women may be less knowledgeable about risks and therefore less able to adopt HIV risk-reducing behaviours. In many parts of rural Africa, this situation is fuelled by inadequate food supplies and malnutrition. [25, 30] This may be exacerbated further by high levels of rural-urban migration.

In 2007, 56% of Zimbabwe’s population were estimated to be living in extreme absolute poverty (on less than US$1 per day). [23] More recent estimates of the proportion living in absolute poverty are lacking but data from the 2012 Zimbabwe Census estimate that 72% of the population are living below the national poverty line.[36, 37] While rates of education and adult literacy remain relatively high compared to the rest of Africa these rates are now also falling, particularly among women. [23] Data from the UNDP Health Development Report for 2013 indicate that between 2006–2010 only 49% of females and 62% of males had at least secondary education and the mean years of schooling is now 7 years.[37] We present data from a population-based survey of 18–22 year olds living in South-Eastern Zimbabwe conducted in 2007 as the final survey of a cluster randomised trial of a community-based multi-component HIV prevention intervention for young rural Zimbabweans. [38, 39] At the time of the survey, Zimbabwe was experiencing extreme economic circumstances, the inflation rate was estimated to be over one million percent with market prices changing rapidly from day to day with multiple exchange rates in operation. The World Food programme estimated that around half of the population at that time were unable to meet their minimum food requirements and were needing assistance to survive.[40] In this context, we explore the relationship between socio-economic position and HIV status among young rural Zimbabwean women and whether different socio-economic domains (asset wealth; ability to afford essential items; and food sufficiency) are associated with HIV risk in different ways.

## Methods

### Study setting and trial design

The community-randomised trial was conducted in 30 rural communities in seven districts in three provinces in south-eastern Zimbabwe. The design of the trial, including sample size calculations, and details of the multi-component intervention are described elsewhere. [38, 39] The analysis reported here is restricted to female participants.

**Fig 1:**
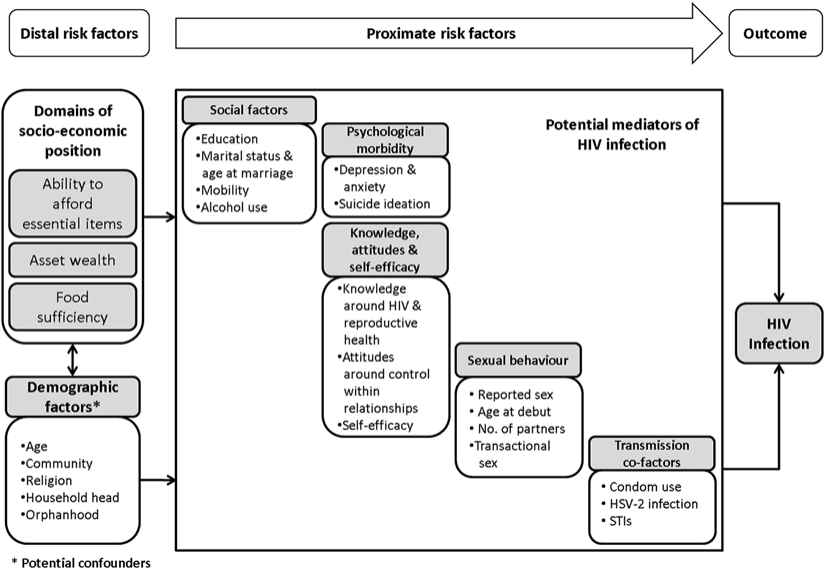
A conceptual framework for the association between domains of socio-economic position and HIV infection in young rural Zimbabwean women.

We hypothesised that poverty might be a cause of vulnerability to HIV among young rural Zimbabweans. We developed a conceptual framework postulating mechanisms by which different socio-economic factors might increase risk of HIV in the rural Zimbabwean context (see Fig. 1). The aim of the framework was to guide data collection and analysis in order to help disentangle the multiple risk factors that function at different levels among young rural Zimbabweans. [28, 41–53].

### Procedures

Six enumeration areas of approximately 100 households were purposively selected in each of the 30 trial communities.[39] All 18–22 year olds who lived in the 180 selected enumeration areas were eligible for inclusion. Households were visited up to two times, after which time an individual was classified as unreachable. Participants provided written informed consent, and completed a self-administered paper-based questionnaire collecting information on factors shown in the framework.

Scales previously validated in South Africa were used to assess attitudes towards control within relationships. [54, 55] Psychological morbidity was assessed using the Shona Symptom Questionnaire (SSQ), a locally validated 14-item screening tool for those at risk of depressive and generalised anxiety disorders. [56] Potential socio-economic indicators were identified through reviewing a number of existing tools including Zimbabwe census questions and questions developed by UNICEF to assess poverty and socio-economic status.[57] In addition, focus group discussions and cognitive interviewing were conducted with community members to ensure that socio-economic indicators included on the questionnaire were contextually appropriate.[58] The final list of indicators incorporated standard UNICEF questions and questions that resulted from the qualitative research and were categorised under the three socio-economic domains identified in the conceptual framework. These were: 1) the ability of the household to afford essential items; 2) household ownership of ‘fixed’ and ‘sellable’ assets; and 3) food sufficiency. Table 1 lists the indicators included under each domain. [59, 60].

**Table 1:** Proportion of participants reporting individual wealth indicators.

**Characteristic**	**N**	**n**	**%**
***Ability to afford essential items:***			
Cannot afford:			
To cook with cooking oil at each meal	2518	1444	57.4
To eat meat/fish at least 4x per week	2478	1823	73.6
To drink tea at least once/day	2480	977	39.4
To use soap to wash our clothes	2484	546	22.0
For each household member to have pair of shoes	2485	1292	52.0
To pay hospital fees if household member sick	2488	1386	55.7
Been absent from school due to no money for fees	2475	1382	55.8
***SEP determined by ability to afford essential items:***	2327		
*SEP1 (wealthiest)*		751	32.3
*SEP2*		727	31.2
*SEP3 (poorest)*		849	36.5
***Ownership of fixed and sellable assets:***			
*Sellable assets—household owns:*			
Oxcart	2518	1111	44.1
Bicycle	2458	901	36.7
Motorcycle	2437	69	2.8
Car	2432	293	12.0
Plough	2475	1889	76.3
Radio	2442	1370	56.1
Television	2429	683	28.1
Telephone	2427	466	19.2
Own any luxury item (motrocycle/car/tv/telephone)	2332	874	37.5
*Fixed assets:*			
Type of toilet:	2572		
Field or bush		679	26.4
Blair (long drop) or Flush toilet		1893	73.6
House built of:	2576		
Traditional materials (poles and dagga) or mud		1983	77.0
Cement or stones		593	23.0
Kitchen Floor:	2584		
Dirt		786	30.4
Cement		1798	69.6
***SEP determined by ownership of fixed and sellable assets:***	2316		
*SEP1 (wealthiest)*		708	30.6
*SEP2*		719	31.0
*SEP3 (poorest)*		889	38.4
***Food sufficiency and security:***			
Sometimes we go to bed hungry	2472	480	19.4
Cannot afford to eat at least 2 meals each day	2480	460	18.6
Adult skipped a meal in last week so food for others	2582	476	18.4
Gone day without food in last week because no food	2586	378	14.6
***Overall reported one or more indicators of insufficient food***	*2577*	*1050*	*40.8*

All participants had a dried blood sample collected which was tested for HIV-1 antibody at the National Microbiology Reference Laboratory in Harare using a validated testing algorithm [61]. All specimens were tested using two ELISA tests (Vironostika HIV Microelisa System BioMerieux, Inc., Durham NC and AniLabsytems EIA kit, AniLabsystems Ltd, Vantaa, Finland), with western blot used in the case of discrepant results. Specimens were further tested for antibodies to HSV-2 using a type-specific HSV-2 assay (Focus HerpeSelect EIA, Focus Technologies, Cypress CA) with the index for diagnosing positive samples raised to >3.4 to minimise false positives. [62]

### Data analysis

Continuous variables were categorised using recognised cut-off values or dichotomised at the median value. Attitudinal and knowledge outcomes were divided into 11 domains, described elsewhere.[39] Binary variables were created identifying those participants who answered all questions correctly in each domain. Participants were classified as orphans if they reported they had lost one or both parents before they were 19 years old. For the mental health SSQ a summed-score of all 14 items was calculated and participants who answered affirmatively to 8 or more of the items were defined as being at risk of common mental disorders. [56]

A variable was created for each socio-economic domain. For sellable assets and essential items, ownership of the asset or ability to afford the item conferred a score of 1; not owning or being able to afford the item was given a score of 0. For ordinal measures, responses were scored on a scale between 0 and 1. For example, type of toilet in the homestead conferred a value of 0 for having to use field or bush, 0.5 for only having access to a neighbour’s toilet, or 1 where the homestead had its own toilet. Indices were created by adding the variable scores in each domain (the essential items summed-score included 7 variables; and the asset ownership score included 11 variables); and then splitting the summed score into three approximately equally sized groups (terciles) from the whole population. As other researchers have found [3], our simple summed-score index was strongly correlated with an index compiled using principal components analysis to weight the items (r≥0.95). Given that weighting indicators appeared to offer little advantage, the simpler summed score approach was used for all analyses. Food sufficiency was determined based on responses to 4 question items (see Table 1). Food insufficiency was defined as an affirmative response to one or more of these questions.

We described the characteristics of the population by level of socio-economic position (SEP). We then examined HIV status by each risk factor identified in the conceptual framework while not including SEP. Age-adjusted odds ratios were calculated and then adjusted (Model 1) for those demographic factors associated with HIV (p≤0.1). Odds ratios were then further adjusted (Model 2) for those risk factors higher in the conceptual framework that were associated with HIV (p≤0.1).

Cox regression (stratified on community) and Kaplan-Meier survival estimates were used to explore time to first marriage and sexual debut by SEP. For other sexual behaviour risk factors and for HIV we used conditional logistic regression to explore associations with SEP, controlling for clustering by community and presenting likelihood ratio tests. Where there was evidence of an association between SEP and HIV after adjusting for age, risk factors that were significant from the first stage of analysis were added to the model using a stepped approach that allowed us to examine how any influence of that socio-economic domain might be explained (‘mediated’) by differences in the more proximate risk factors.

Finally we explored the relationship between SEP and sexual behaviour using the same approach as described above. We created a binary composite variable for sexual risk by combining several sexual risk behaviour variables. ‘High sexual risk’ females were those who reported two or more of the following characteristics: sexual debut at 17yrs or younger; 2 or more lifetime partners; any partner 6 or more years older; and not using a condom at last sex. All other women, including those who reported that they had never had sex, were classified as ‘low sexual risk’.

### Ethical approval

The trial and all research related to the trial was approved by the Medical Research Council of Zimbabwe and the ethics committees of University College London Hospitals and the London School of Hygiene & Tropical Medicine.

## Results

Of 4822 eligible individuals identified at households, 4672 (97%) participated in the survey and were included in the analyses, 2593 (56%) of whom were female. The median age of female participants was 19 years. Just over 70% had completed ≥3 years of secondary education. Using categorical variables for marital status and age at first marriage, almost half (45%; 1175) were either currently or previously married; just under 60% of married females were married before they were 18 years old; 18% were married aged 16 or younger. Rates of orphaning were high; 47% (1087/2509) reported having lost one or both parents; 24% had lost their father, 7% their mother, and 16% had lost both parents.

### Distribution of socio-economic indicators

Table 1 shows the proportion of female participants reporting individual socioeconomic indicators. There was a high level of correlation between all binary and ordinal poverty indicators and evidence of increased food insufficiency with decreasing socio-economic position as determined by the other SEP indices (chi-square test for trend p<0.001). For example 62% of those in the poorest socio-economic position defined by ability to afford essential and everyday items, reported insufficient food compared to 19% of those in the wealthiest category and 37% in the middle group. Similar proportions of food insufficiency were seen among SEP terciles defined by asset ownership (20% reported food insufficiency in the wealthiest tercile vs. 39% in the mid tercile and 60% in the poorest asset ownership group.

### Socio-economic position and potential risk factors for HIV infection

Table 2 presents characteristics and potential risk factors for female participants by socio-economic position. Older females were more likely to report being poor (by all definitions of SEP) than younger women. Poorer women were more likely to live in female-headed households and less likely to live in a household with an older or more educated household head. Orphaning was more common among those of lower SEP. Educational attainment was strongly associated with each socio-economic domain with poorer females significantly less likely to have had any secondary education. Poorer women were significantly more likely to be married (test-for-trend p-value<0.001) and to be married younger.

**Table 2:** Characteristics of female participants by socio-economic position (SEP) and SEP domain.

	**Domain of socio-economic position (SEP)**											
	**Ability to afford essential items**					**Asset wealth**					**Food sufficiency**		
**Characteristic**	SEP1^1^		SEP2	SEP3^2^	p-value^3^	SEP1^1^	SEP2	SEP3^2^	p-value^3^	Food sufficient	Food insufficient	p-value^3^
	**N**	**(%)^4^**	**(%)^4^**	**(%)^4^**		**(%)^4^**	**(%)^4^**	**(%)^4^**		**(%)^4^**	**(%)^4^**	
	751	32.3	31.2	36.5		30.6	31.0	38.4		59.2	40.8	
***Demographic:***												
*Age:*												
18 years	287	38.2	38.9	32.9		38.4	39.2	32.3		38.2	35.0	
19–20 years	245	32.6	28.6	31.6		32.2	31.8	29.6		31.6	29.3	
21–22 years	219	29.2	32.5	35.6	p = 0.018	29.4	28.9	38.1	p<0.001	30.3	35.7	p = 0.014
*Religion:*												
Catholic	164	21.9	17.9	14.5		21.4	17.5	15.4		19.5	16.4	
Protestant	218	29.2	26.8	22.2		32.3	25.6	19.8		26.7	25.0	
Apostolic/pentecostal	229	30.7	33.9	40.4		27.5	36.6	41.2		34.1	36.8	
Other	126	16.9	18.3	21.0		17.4	18.9	20.6		18.2	19.1	
None	10	1.3	3.1	1.8	p<0.001	1.4	1.4	2.9	p<0.001	1.5	2.8	p = 0.031
*Distance from tar road:*												
<15km	265	35.3	33.7	31.4		35.4	33.2	31.8		35.2	31.4	
15–30km	306	40.7	36.6	37.3		39.1	36.0	38.4		38.3	37.4	
>30km	180	24.0	29.7	31.2	p = 0.020	25.4	30.7	29.8	p = 0.158	26.5	31.1	p = 0.022
*Household head:*												
Female	211	28.3	34.3	38.5	*p<0.001*	28.7	34.6	36.8	*p<0.001*	32.3	36.7	p = 0.019
40 years or older	424	56.7	55.6	54.2	p = 0.612	59.5	57.8	50.6	*p<0.001*	58.9	46.2	p<0.001
Some primary or secondary education	658	97.6	93.2	89.8	*p<0.001*	96.2	95.8	88.8	*p<0.001*	95.1	90.2	p<0.001
*Orphanhood:*												
Orphan (lost one or both parents)	279	37.9	47.4	55.8	*p<0.001*	39.6	47.6	52.5	*p<0.001*	42.0	55.2	p<0.001
***Social Factors:***												
*Education:*												
Secondary education (1 year or more)	690	91.9	86.7	81.3	*p<0.001*	94.8	89.0	77.9	*p<0.001*	90.1	78.4	p<0.001
*Marital status:*												
Currently/have been married	276	36.9	47.2	49.5	*p<0.001*	34.0	42.6	54.2	*p<0.001*	39.6	54.1	p<0.001
Median age at first marriage (years)		21.5	20.5	20.5	p<0.001	22.5	21.5	20.5	p<0.001	21.5	20.5	p<0.001
*Time in community:*												
5 years or more	343	50.1	57.5	64.7		49.5	60.0	63.9		56.1	59.8	
1–4 years	104	15.2	11.2	11.3		14.0	11.3	12.0		12.9	11.4	
1 year or more but not continuously	65	9.5	11.5	9.2		10.5	10.0	8.4		10.4	9.8	
Less than a year or visiting	173	25.3	19.8	14.8	p<0.001	26.0	18.7	15.7	p<0.001	20.5	19.0	p = 0.361
*Alcohol consumption:*												
Drunk alcohol in last 4 weeks	165	22.2	23.7	25.9	p = 0.218	22.2	21.0	27.5	*p = 0.010*	22.3	29.0	p<0.001
***Psychological morbidity and suicide ideation:***												
Reported 8 or more symptoms of depression & anxiety	389	62.7	71.3	76.2	*p<0.001*	67.4	69.3	73.2	p = 0.068	65.6	78.4	p<0.001
Suicidal ideation in last week	218	30.2	33.6	38.3	*p<0.001*	32.9	30.3	37.8	*p = 0.029*	27.7	45.9	p<0.001
***Knowledge, attitudes & self-efficacy:***												
*Knowledge:*												
HIV acquisition (3 questions)^5^	139	18.5	18.8	18.6	p = 0.986	18.2	19.5	18.3	p = 0.793	17.7	19.4	p = 0.256
STI acquisition (2 questions)^5^	333	44.3	37.4	36.3	*p = 0.001*	44.4	36.7	37.0	*p = 0.004*	40.5	34.8	p = 0.003
Pregnancy prevention (2 questions)^5^	274	36.5	28.1	28.2	*p<0.001*	34.8	31.6	27.4	*p = 0.001*	32.8	24.3	p<0.001
*Attitudes around control over sex:*												
Responded to ≥7 questions correctly (10 questions)	405	58.7	49.3	53.4	*p = 0.062*	62.7	50.8	49.2	*p<0.001*	59.0	43.8	p<0.001
Control around sexual refusal (3 questions)^5^	205	28.0	21.7	25.8	*p = 0.351*	24.9	27.8	23.4	*p = 0.425*	26.9	21.9	p = 0.005
Control around sexual partners (4 questions)^5^	263	37.0	30.2	31.3	*p = 0.020*	37.7	32.4	29.8	*p = 0.001*	36.8	24.3	p<0.001
Control around safe sex (2 questions)^5^	278	38.0	33.1	33.2	*p = 0.051*	41.2	33.1	32.2	*p<0.001*	37.8	29.4	p<0.001
*Self-efficacy:*												
Condom self-efficacy (3 questions)^5^	464	62.4	63.5	65.0	p = 0.570	66.9	62.2	62.4	p = 0.104	63.7	62.4	p = 0.508
Sexual refusal self-efficacy (2 questions)^5^	535	72.1	68.5	70.4	p = 0.324	75.2	70.2	67.3	*p<0.001*	73.0	61.2	p<0.001
HIV testing self-efficacy (3 questions)^5^	535	71.7	69.4	70.2	p = 0.620	74.2	70.6	68.4	*p = 0.011*	72.6	64.3	p<0.001
***Sexual risk factors^5^:***												
Reported (vaginal) sex	327	44.7	53.8	58.2	*p<0.001*	45.2	48.0	61.5	*p<0.001*	47.4	61.2	p<0.001
Sexual debut younger than 18 years	125	17.1	24.9	26.9	*p<0.001*	15.7	22.4	28.0	*p<0.001*	18.2	31.8	p<0.001
Median age at sexual debut (years)		20.5	19.5	19.5	p<0.001	20.5	20.5	19.5	p<0.001	20.5	19.5	p<0.001
2 or more lifetime partners	72	9.8	10.2	13.5	*p = 0.021*	10.0	9.8	13.1	p = 0.060	9.3	14.0	p = 0.001
Had a sexual partner who was >5 years older	137	18.7	22.6	26.2	*p<0.001*	20.9	19.7	26.9	*p = 0.004*	20.0	28.0	p<0.001
Had sex for material/financial support	70	9.6	9.4	12.5	p = 0.081	7.9	8.3	13.2	*p<0.001*	7.9	16.8	p<0.001
***Transmission co-factors:***												
Use condoms inconsistently or never use^5^	324	44.3	52.4	58.2	*p<0.001*	44.3	47.7	61.0	*p<0.001*	46.8	60.7	p<0.001
No condom used at last sex^5^	244	33.6	41.9	44.8	*p<0.001*	33.3	37.3	48.8	*p<0.001*	36.2	47.0	p<0.001
Reported one or more symptoms of STD	217	29.0	33.0	38.1	*p<0.001*	29.3	31.1	38.8	*p<0.001*	30.6	40.8	p<0.001
HSV-2 positive	74	10.1	11.1	11.3	p = 0.713	5.6	8.8	12.8	*p = 0.004*	9.1	14.3	p<0.001

^1^ Wealthiest category (SEP1);

^2^ Poorest category (SEP3);

^3^ P-value from Chi-square test, Mantel-Haenszel test-for-trend (*given in italics where chi-square p*≤0.05) or Cox proportional hazard depending on type of data;

^4^ Column percentages (%);

^5^ Reference category includes those who have never had sex.

The proportion of participants at risk of common mental disorders increased as socio-economic position decreased; 46% of females who had insufficient food reported considering suicide at some point in the previous week, compared to 28% of those who had not had to go without food. However, there was no evidence of association between SEP and knowledge of HIV acquisition for any of the socio-economic domains. Attitudes around control within relationships were strongly associated with food sufficiency status with food insufficient women reporting less control. The lowest levels of self-efficacy were also seen among this group of women.

### Risk factors for HIV infection

Overall HIV prevalence was 7.7% (95%CI: 6.7–8.7) and there was increasing HIV risk with increasing age (Table 3). After adjusting for age the only demographic factor significantly associated with HIV infection was the age of the household head. Of the social factors (see Fig. 1), education, marital status, age of first marriage and alcohol consumption were all strongly associated with HIV infection even after adjusting for age and age of household head. Having some secondary education was associated with lower HIV prevalence, whereas ever being married was associated with a higher prevalence of HIV infection. Alcohol consumption in the last 4 weeks was also significantly associated with higher prevalence of HIV. After further adjusting for all demographic and social variables in Model 2, there was weak evidence that education was associated with reduced risk of HIV infection but there continued to be strong evidence of increased risk associated with marital status.

**Table 3:** Risk factors for HIV infection excluding socio-economic position.

**Characteristic**	**Proportion HIV positive**		**Age-adjusted OR^a^ [95% CI]**		**Age-adjusted Model 1^1, a^**		**Age-adjusted Model 2 (n = 2022)^2, a^**	
	**n/N**	**%**	**OR**	**[95% CI]**	**OR**	**[95% CI]**	**OR**	**[95% CI]**
***Demographic:***									
*Age:*				*p<0.001*		*p<0.001*		
18 years	21/953	2.2	1.00		1.00			
19–20 years	62/794	7.8	3.74	[2.25–6.21]	4.09	[2.38–7.03]		
21–22 years	116/841	13.8	7.13	[4.41–11.51]	7.57	[4.52–12.66]		
*Age of household head:*				*p = 0.004*		*p = 0.003*		
Under 40 years old	115/1191	9.7	1.00					
40 years or older	81/1374	5.9	0.64	[0.47–0.86]	0.62	[0.45–0.85]		
***Social Factors:***								
*Education:*				*p = 0.002*		*p = 0.012*		*p = 0.066*
None/primary	42/380	11.0	1.00		1.00		1.00	
Secondary or higher education	154/2194	7.0	0.54	[0.37–0.78]	0.60	[0.40–0.88]	0.63	[0.39–1.02]
*Marital status:*				*p<0.001*		*p<0.001*		*p<0.001*
Never	39/1403	2.8	1.00		1.00		1.00	
Currently/have been married	160/1176	13.6	3.86	[2.62–5.69]	3.86	[2.58–5.77]	2.82	[1.75–4.53]
*Age first married:*				*p = 0.017*		*p = 0.041*		*p = 0.433*
17 or older/never married	143/2168	6.6	1.00		1.00		1.00	
16 or younger	23/169	13.9	1.88	[1.15–3.06]	1.73	[1.04–2.85]	1.27	[0.70–2.30]
*Alcohol drunk in last 4 weeks:*				*p = 0.001*		*p = 0.002*		*p = 0.219*
Never drunk alcohol/no alcohol last 4 wks	127/1916	6.6	1.00		1.00		1.00	
Drunk alcohol in last 4 weeks	68/638	10.7	1.76	[1.27–2.43]	1.72	[1.24–2.39]	1.30	[0.86–1.98]
***Psychological Morbidity:***								
*Suicide ideation in last week:*				*p = 0.022*		*p = 0.059*		*p = 0.075*
Never considered suicide	101/1546	6.5	1.00		1.00		1.00	
Sometimes/always feel like committing suicide	78/835	9.3	1.46	[1.06–2.00]	1.37	[0.99–1.89]	1.21	[0.82–1.78]
***Knowledge, attitudes & self-efficacy:***								
*Knowledge:*								
*Around pregnancy prevention (2 questions):*				*p = 0.025*		*p = 0.032*		*p = 0.006*
Low	150/1829	8.2	1.00		1.00		1.00	
Both question responses ‘correct’	48/756	6.4	0.68	[0.48–0.96]	0.69	[0.48–0.98]	0.61	[0.40–0.94]
*Attitudes around control over sex*								
*Control around sexual partners (4 questions):*				*p = 0.090*		*p = 0.051*		*p = 0.038*
Low	107/1612	6.6	1.00		1.00		1.00	
All 4 question responses ‘correct’	68/751	9.0	1.33	[0.96–1.85]	1.40	[1.00–1.95]	1.46	[0.98–2.18]
*Self-efficacy:*								
*Condom self-efficacy (3 questions)*				*p = 0.004*		*p = 0.003*		*p = 0.049*
Poor	45/941	4.8	1.00		1.00		1.00	
All 3 question responses ‘correct’	152/1612	9.4	1.65	[1.16–2.36]	1.71	[1.19–2.45]	1.38	[0.89–2.14]
***Sexual Risk Factors:***								
*Reported vaginal sex:*				*p<0.001*		*p<0.001*		*p = 0.487*
Never had sex/never had vaginal sex	28/1175	2.4	1.00		1.00		1.00	
Yes	165/1326	12.4	3.87	[2.51–5.97]	3.99	[2.54–6.28]	1.31	[0.61–2.83]
*Sexual debut (vaginal sex):*				*p<0.001*		*p<0.001*		*p = 0.447*
Never had sex/18 or older	121/1910	6.3	1.00		1.00		1.00	
Younger than 17 years	72/591	12.2	2.07	[1.50–2.85]	2.02	[1.45–2.81]	1.19	[0.76–1.86]
*Number of partners:*				*p<0.001*		*p<0.001*		*p = 0.383*
Never had sex/Only 1 lifetime partner	141/2223	6.3	1.00		1.00		1.00	
Two or more lifetime partners	52/278	18.7	2.34	[1.63–3.38]	2.37	[1.64–3.43]	1.25	[0.76–2.08]
*Age of partners:*				*p<0.001*		*p<0.001*		*p = 0.060*
Never had sex/partners same age (+/-5 yrs) or younger	101/1924	5.2	1.00		1.00		1.00	
Any partner 6–10 yrs older	56/373	15.0	2.45	[1.70–3.52]	2.51	[1.73–3.63]	1.48	[0.92–2.37]
Any partner >10 yrs older	36/204	17.6	3.09	[1.99–4.80]	3.21	[2.06–5.01]	1.89	[1.07–3.33]
*Transactional sex:*								
*Had sex with partner for material/other support:*				*p<0.001*		*p<0.001*		*p = 0.170*
Never had sex/never had transactional sex	148/2214	6.7	1.00		1.00		1.00	
Yes	45/287	15.7	2.34	[1.60–3.42]	2.26	[1.54–3.32]	1.45	[0.86–2.46]
***Transmission co-factors:***								
*Condom use:*								
*Used condom at last sex with last partner:*				*p = 0.005*		*p = 0.012*		*p = 0.046*
Never had sex/used a condom at last sex	75/1475	5.1	1.00		1.00		1.00	
Did not use a condom at last sex	117/1011	11.6	1.59	[1.14–2.21]	1.55	[1.10–2.17]	0.58	[0.34–0.98]
*Reported condom use with any partner:*				*p<0.001*		*p<0.001*		*p = 0.312*
Never had sex/always used a condom with all partners	29/1189	2.4	1.00		1.00		1.00	
Only sometimes/never used condoms with any partner	164/1312	12.5	3.80	[2.48–5.83]	3.90	[2.50–6.09]	1.47	[0.70–3.10]
*Sexually transmitted infections:*								
*Reported any symptom of STI:*				*p = 0.001*		*p = 0.004*		*p = 0.387*
No	103/1671	6.2	1.00		1.00		1.00	
Yes	92/891	10.3	1.65	[1.22–2.24]	1.58	[1.16–2.16]	1.20	[0.80–1.80]
*Biomarker for HSV-2 infection:*				*p<0.001*		*p<0.001*		*p<0.001*
Negative	75/2226	3.4	1.00		1.00		1.00	
Positive	99/279	35.5	12.83	[8.91–18.47]	12.24	[8.43–17.77]	8.60	[5.57–13.27]

^1^ Adjusted for age and age of household head.

^2^ Adjusted for age and age of household head and other variables higher in conceptual framework (Fig. 2) that remain associated (at p≤0.1) with HIV after adjustment.

^a^ Likelihood ratio p-value.

**Fig 2:**
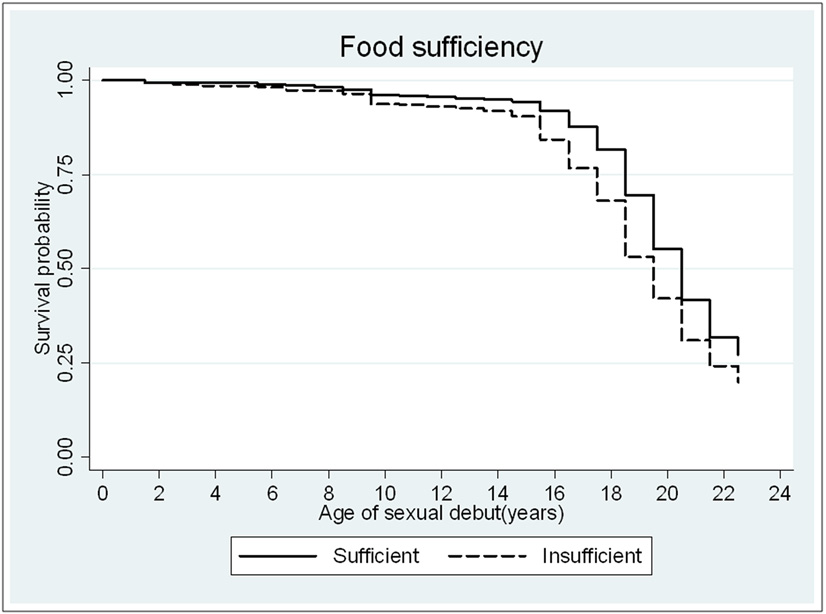
Survival time to age at sexual debut by food sufficiency status.

After adjustment for potential confounders and those social factors associated with HIV infection there was little evidence that psychological morbidity was associated with HIV. Only three of the knowledge and attitudinal outcomes were associated with HIV infection, although the direction of effect differed depending on the item (see Table 3). After adjusting for age and other potential confounding factors (Model 1) there was strong evidence of an association between reported sexual behaviours and increased risk of HIV infection. However, after further adjusting for those factors higher in the conceptual framework that were associated with HIV (Model 2), there remained only a borderline significant association with age of sexual partners. Not using a condom during last sexual encounter was initially associated with a higher prevalence of HIV (see age-adjusted and adjusted OR in Model 1); however controlling for other factors in Model 2 led to a change in the direction of effect with lack of condom use at last sex appearing now to be associated with a reduced risk of HIV suggesting an association with one of the other proximate risk factors or some unmeasured factor. HSV-2 infection was strongly associated with HIV infection.

### Socio-economic position and HIV infection

The prevalence of HIV varied little between SEP categories for the SEP domains defined by ability to afford essential items and ownership of fixed and sellable assets (Table 4). However, there was evidence of an association between food sufficiency and HIV infection (aOR 1.6). Further adjusting for social factors associated with HIV (Model 3) led to a fall in the OR to 1.4, suggesting that differences in these social factors between women with sufficient and insufficient food partly explained the difference seen in HIV prevalence. The OR fell further as the model was adjusted for knowledge, attitudes and self-efficacy variables (Model 4), for sexual risk factors (Model 5) and finally for transmission co-factors (Model 6), with the final adjusted OR being 1.27 (95% CI: 0.84–1.94). Each fall in OR suggests that these proximate factors mediated the relationship between food sufficiency and HIV among these female participants.

**Table 4:** Socio-economic position as a risk factor for HIV infection among females by socio-economic domain.

**Characteristic**	**Proportion HIV positive**		**Age-adjusted OR [95% CI[**		**Adjusted OR Model 3 ^1^**		**Adjusted OR Model 4 ^2^**		**Adjusted OR Model 5 ^3^**		**Adjusted OR Model 6 ^4^**	
	**n/N**	**%**	**OR**	**[95% CI]**	**OR**	**[95% CI]**	**OR**	**[95% CI]**	**OR**	**[95% CI]**	**OR**	**[95% CI]**
***Domains of socio-economic position (SEP)***												
*SEP defined by ability to afford essential items:*				*p = 0.926*								
SEP 1 (Wealthiest)	52/748	6.9	1.00									
SEP 2	51/727	7.0	0.92	[0.57–1.47]								
SEP 3 (Poorest)	68/847	8.0	0.99	[0.64–1.53]								
*SEP defined by ownership of fixed & sellable assets:*				*p = 0.839*		**						
SEP 1 (Wealthiest)	47/707	6.6	1.00									
SEP 2	44/717	6.1	0.95	[0.58–1.55]								
SEP 3 (Poorest)	76/887	8.6	1.09	[0.69–1.70]								
*Food sufficiency & security:*				*p = 0.009*		*p = 0.079*		*p = 0.082*		*p = 0.113*		*p = 0.261*
Food sufficient	92/1524	6.0	1.00									
Food insufficient ^5^	104/1049	9.9	1.64	[1.13–2.36]	1.40	[0.96–2.04]	1.41	[0.96–2.08]	1.37	[0.93–2.03]	1.27	[0.84–1.94]

^1^ Adjusted for age, age of household head, education, marital status & alcohol consumption.

^2^ Adjusted for variables in model 3 & knowledge around pregnancy prevention, attitudes around sexual partners & condom self-efficacy.

^3^ Adjusted for variables in model 4 & age of sexual partners.

^4^ Adjusted for variables in model 5 & condom use at last sex & HSV-2 infection.

^5^ Food insufficiency—reported adult skipping meals and/or going day without food; going to bed hungry; <2 meals per day.

### Determinants of risky sexual behaviour

There was strong evidence of association between SEP and most aspects of reported sexual behaviour, in all three socio-economic domains (Table 3). Poorer women were more likely to report ever having had (vaginal) sex; to have experienced earlier sexual debut; to have had more partners and partners who were 6 or more years older and to report having had sex for material or financial support. Women who reported having insufficient food reported earlier sexual debut (age adjusted HR = 1.49; 95% CI: 1.33–1.66—see Fig. 2) and were more likely to report sex for material or financial support (age-adjusted OR = 2.30; 95% CI: 1.78–2.98).

Rates of reported condom use were generally low, only 59.6% of all women reported using a condom at last sex (vaginal, anal, consensual or non-consensual). Poorer women were significantly less likely to report condom use than women in the wealthier SEP categories for all 3 socio-economic domains. There was evidence of increasing prevalence of reported symptoms of STIs with declining SEP. Women who had insufficient food were more likely to be HSV-2 positive (aOR 1.7 95% CI: 1.30–2.20).

There was also a significant association seen between SEP and reporting two or more high-risk sexual behaviours among these young women (Table 5), even after adjusting for age, with those women categorised in the poorest categories of each socio-economic domain more likely to report high-risk behaviours than those in the wealthiest categories. For example, women reporting insufficient food were more likely to report two or more high-risk behaviours than women with sufficient food supply (aOR 1.6; 95% CI: 1.3–2.0). These associations remained significant after adjusting for potential confounding factors although there was a slight fall in the ORs for the association in each case (Model 1). After adjusting for those social factors that were associated with reporting risky sexual behaviour (Model 2), the OR for the association between SEP and risky behaviour fell for all three socio-economic domains suggesting that the influence of asset wealth on higher risk behaviours was explained in part through differences in educational attainment, marital status and alcohol consumption between these SEP groups. Adjusting these estimates further by incorporating psychological, knowledge, attitudinal and self-efficacy variables in the model (Model 3) led to little change in the OR for risky behaviour associated with food sufficiency.

**Table 5:** Socio-economic position as a risk factor for reporting risky sexual behaviour by socio-economic domain.

	**Prevalence of ‘high risk’ behaviour ^1^**		**Age-adjusted OR**		**Adjusted OR Model 1 ^2^**		**Adjusted OR Model 2 ^3^**		**Adjusted OR Model 3 ^4^**	
	**n**	**%**	**OR**	**[95% CI]**	**OR**	**[95% CI]**	**OR**	**[95% CI]**	**OR**	**[95% CI]**
***Domains of Socio-economic position (SEP):***										
*SEP defined by ability to afford essential items:*				*p = 0.009*		*p = 0.030*		*p = 0.547*		*p = 0.594*
SEP 1 (Wealthiest)	198	26.4	1.00		1.00		1.00		1.00	
SEP 2	238	32.7	1.38	[1.04–1.81]	1.35	[1.02–1.78]	1.05	[0.77–1.43]	1.04	[0.76–1.43]
SEP 3 (Poorest)	313	36.9	1.49	[1.14–1.94]	1.40	[1.07–1.84]	1.18	[0.87–1.60]	1.16	[0.86–1.59]
*SEP defined by ownership of fixed & sellable assets:*				*p = 0.014*		*p = 0.042*		*p = 0.467*		*p = 0.584*
SEP 1 (Wealthiest)	192	27.1	1.00		1.00		1.00		1.00	
SEP 2	217	30.2	1.07	[0.81–1.41]	1.04	[0.79–1.38]	0.82	[0.60–1.13]	0.84	[0.61–1.16]
SEP 3 (Poorest)	332	37.4	1.43	[1.10–1.87]	1.36	[1.04–1.78]	0.88	[0.64–1.20]	0.90	[0.66–1.23]
*Food sufficiency & security:*				*p<0.001*		*p<0.001*		*p = 0.044*		*p = 0.072*
Food sufficient	424	27.8	1.00		1.00		1.00		1.00	
Food insufficient^5^	419	39.9	1.60	[1.29–1.98]	1.51	[1.21–1.88]	1.29	[1.01–1.66]	1.26	[0.98–1.63]

^1^ ‘High risk’ behaviour defined as 2 or more risky behaviours: sexual debut ≤17yrs; ≥2 lifetime partners; any partner ≥6 years older; not using a condom at last sex.

^2^ Adjusted for age, age of household head & orphanhood.

^3^ Adjusted for variables in model 1 & education, marital status & alcohol consumption.

^4^ Adjusted for variables in model 2, risk of depression & anxiety, suicide ideation, attitudes around sexual refusal and sexual partners and condom self-efficacy.

^5^ Food insufficiency—reported adult skipping meals and/or going day without food; going to bed hungry; <2 meals per day.

## Discussion

Among young rural Zimbabwean women, indicators of lower socio-economic position were associated with lower educational attainment, earlier marriage, higher rates of symptoms of depression and anxiety and suicidal ideation. Food insufficiency was associated with lower levels of HIV knowledge, less strong attitudes around sexual control and poorer self-efficacy. Lower SEP was also associated with high-risk sexual behaviours. In particular, insufficient food was strongly associated with increased reporting of these behaviours and was also strongly associated with lack of condom use, reporting ever had symptoms of STIs and HSV-2 infection. The more robust relationship between food insufficiency and sexual risk than the other SEP domains investigated here, may reflect the temporal nature of those domains which could have been influenced by the Zimbabwean economy and HIV epidemic within Zimbabwe.[58] It is not possible from this study to determine how socio-economic position has changed over time and it is possible that food insufficiency is more of a reflection of current wealth whereas asset ownership may reflect economic wealth in the recent past. Likewise with the domain ‘ability to afford essential items’, whilst several of the items used to assess this e.g. ability to afford cooking oil and tea, likely reflect the level of current disposable income, owning shoes and absenteeism from school due to lack of fees are more likely to indicate poverty over a longer period of time. It may also be that being poor itself does not drive high-risk behaviours, but being poor to the point of having insufficient food and being hungry, or having hungry children in a household, instigates a need among these women to do whatever is required to survive including disregarding their own safety and health. The relationship between food insufficiency and high risk sexual behaviours was further supported by some evidence of an association between food insufficiency and HIV prevalence among these women. This relationship appeared to be explained in part through differences in social factors such as marriage, education and alcohol use, and in more proximate risk factors such as psychological morbidity, knowledge, attitudes, self-efficacy and sexual behaviour.

This study had a number of strengths, including a high response rate and large sample size. We also included data on a range of lifestyle and economic indicators, sexual behaviour and biomarkers for both HIV and HSV-2. This enabled us to explore a number of hypothesised relationships outlined in the conceptual framework, whilst incorporating different aspects of poverty and other factors (e.g. psychological morbidity).

A key limitation of the study was that the data were cross-sectional. It was therefore not possible to infer the temporality of the associations seen here. Where associations were seen, we could not assess whether poverty increased risk of HIV infection, or, whether risk poverty arose as a consequence of HIV infection. A further potential limitation relates to the validity of reported sexual behaviour data, an issue affecting all sexual behaviour research among young people in Africa. [49, 63–69] While we implemented best-practice approaches to the collection of these data, limitations in their validity may remain. Measuring income and/or expenditure is generally considered the gold standard measure of socio-economic position, while we measured assets, ability to pay and food security. While our approach again has limitations, at the time of the survey market prices fluctuated radically and rapidly, inflation was in excess of one million percent and there were multiple exchange rates in operation, making any kind of monetary monitoring almost impossible. [70] While the proportion of participants reporting food insufficiency seems high, World Food Programme estimates of the number of people requiring food aid in Zimbabwe at that time also suggest high levels of food insecurity. [40] Despite inflation stabilising, the position regarding food security has not changed significantly since the study was conducted. Recent estimates suggest that only 49% of Zimbabweans were food secure in 2012. [71] The World Food programme predictions of the number of people who will be food insecure in the next year continue to increase with an estimated 2.2 million people (one in four of the rural population), expected to need food assistance in the next year. [72]

While there were associations between asset wealth and sexual behaviour, in contrast with some previous studies [10, 26, 73], we found no association between either asset wealth or ability to afford essential items and HIV prevalence among this population. As discussed earlier, the populations most at risk tend to change over time. [14, 25] Between 1998 and 2003 HIV incidence was associated with asset wealth among young men in Manicaland, but there was no association between wealth index and incidence among young women. [3] Some studies have used alternative indicators of socio-economic position to those used here such as household income, education and occupation. [14, 74]. We found educational attainment to be strongly correlated with socio-economic position, and it was associated with lower prevalence of HIV and less reporting of risky sexual behaviours. These results concur with results from other studies that have demonstrated a protective effect of education on sexual behaviour and/or on HIV prevalence. [3, 27, 28, 32, 75–78]

The study data did suggest an association between food insecurity, our third domain of socioeconomic position, and HIV prevalence. Few studies have explored how food insufficiency contributes to the spread of HIV although the likelihood and plausibility of the association have frequently been discussed. [12, 15, 18, 29, 30, 33, 34, 79] A study in Botswana and Swaziland found similar results to those reported here with food insufficiency among women significantly associated with increased HIV risk behaviour although no biomarkers of risk were collected in that study. [30] Qualitative research conducted in southern Zambia by Bryceson et al. demonstrated how participants believe that hunger is a driver of the HIV epidemic, particularly for women. [80] More recently Tsai et al. reported on a study of sexually active women in Brazil which demonstrated that severe food insecurity was associated with reduced odds of condom use and an increase in potential symptoms of STIs. Of note, severe food insecurity was associated with increased risk taking even after controlling for household wealth and did not appear to be mediated either by gender power inequity or poor nutrition. The authors concluded that lack of food security appeared to have a direct association with sexual risk taking which would appear to support the relationship seen in this study between food insufficiency and HIV risk behaviours. Furthermore, a project conducted in Malawi, giving cash transfers to young women showed a reduction in risk of HIV infection among these women associated with the relatively small increase in economic security.[81, 82] The influence of food security as a driver for adopting risky sexual behaviours among these young Zimbabwean women is important particularly in light of the current level of food aid that appears to be needed within the country. [40, 72] The importance of food insecurity is increasingly being recognised as a variable of central importance in HIV prevention efforts and the integration of nutrition and HIV/AIDS programming activities is more and more frequently discussed and proposed. [18, 81–85]

The extreme economic circumstances that Zimbabwe endured at the time of this study may influence the comparability of the results to other populations. However, the turmoil experienced by Zimbabwe over recent years is not unique in Africa. Given the paucity of research that explores the link between poverty and HIV during times of rapid economic contraction, this study provides an important and relevant addition to this field of research. The overall decline in HIV infection within Zimbabwe during a time when the levels of poverty, particularly food insufficiency, and unemployment were undeniably increasing, challenges the idea that poverty was driving the overall epidemic trajectory in Zimbabwe over this period [4, 86, 87]. However, the evidence presented here suggests that the poorest young women, particularly those who did not have access to sufficient food, had riskier sexual behaviour and were at increased risk of HIV and HSV-2.

The increased prevalence of high-risk behaviours and HIV in those young women of lower socio-economic position is a cause for concern. Programme planners need to ensure that those made vulnerable through increasing poverty and food insufficiency are successfully and appropriately targeted. There are many practical and ethical difficulties in determining how to deliver food to those most at risk amidst widespread food insecurity. Interventions to tackle poverty and keep young people in school will not be successful without a high level of governmental support. [12] The challenge now is how to rebalance these social and economic inequalities, particularly among young rural women. Structural interventions that include cash transfers, vocational skills training and microfinance interventions are increasingly being considered as important components of the HIV prevention armoury but further exploration in a range of contexts is required, and the reality is that no single or simple intervention will be sufficient. [8, 18, 33, 82, 88–91]

In summary, this study provides further evidence that young poor rural women in Zimbabwe, particularly those who do not have access to sufficient food and those with lower educational attainment, were at increased risk of HIV infection in 2007 and are likely to remain so given that many of these risk factors continue to be prevalent within the country. It is evident that poverty is associated with many factors that drive and influence HIV risk behaviours. However, the notion that ‘HIV is a disease caused by poverty’ is, as others have concluded, too simplistic. [7, 11, 13] These data suggest that targeted structural interventions that tackle social and economic constraints are likely to be a critical component of combination HIV prevention programming and policy.
